# Medicaid Accessibility for Ophthalmologists and Optometrists in Florida: A Pilot Study

**DOI:** 10.7759/cureus.90437

**Published:** 2025-08-18

**Authors:** Darby D Miller, Isabella V Wagner, Michael W Stewart, Abhimanyu S Ahuja, Syril Dorairaj, Richard D Ten Hulzen, Aaron Y Lee

**Affiliations:** 1 Ophthalmology, Mayo Clinic, Jacksonville, USA; 2 Ophthalmology, Casey Eye Institute, Portland, USA; 3 Ophthalmology, Washington University School of Medicine, St. Louis, USA

**Keywords:** medicaid, medicaid access, medicaid services, ophthalmologist, optometrist

## Abstract

Objective and methods: This study aimed to evaluate Medicaid accessibility for ophthalmologists and optometrists in Florida. A cross-sectional telephone survey was conducted between February and March 2018 using a geographically uniform sample of 22 ophthalmologists and 80 optometrists registered with Medicaid. Providers were asked whether they accepted new Medicaid patients (adult and/or pediatric) and for the earliest available appointment. Categorical variables were compared using Fisher’s exact test, and time to next appointment was analyzed using the Wilcoxon rank-sum test (α = 0.05).

Results: Across all age groups, a greater proportion of ophthalmologists (95.4%) accepted some form of Medicaid compared to optometrists (83.8%) (p=0.29), a higher percentage of ophthalmologists reported accepting new Medicaid patients (86.4% vs. 76.3%; P=0.39) and a slightly higher proportion of ophthalmologists reported they would take adult patients with some kind of Medicaid insurance (90.9% vs. 85.0%; p=0.73). Weekend availability was slightly higher among ophthalmologists (63.6% vs. 56.3%; p=0.63). Acceptance of new pediatric Medicaid patients and time to next appointment were similar between provider groups (p=1.00 and p=0.74, respectively).

Conclusion: A greater proportion of ophthalmologists reported accepting adult Medicaid patients and offering weekend availability compared to optometrists, although differences were not statistically significant. Larger studies are needed to confirm these trends.

## Introduction

In the United States, more than four million adults aged 40 years and older are visually impaired or blind, and this is projected to more than double by 2050 [1,2]. Visual impairment, which affects approximately 3% of children under the age of 18, is recognized as a leading developmental disability [3,4]. Though early detection of vision loss with timely intervention is critical to preventing long-term visual disability, significant barriers to routine eye care persist [2]. A major barrier to healthcare access, socioeconomic status (SES), has been strongly associated with a higher prevalence of vision-threatening comorbidities and a diminished vision-related quality of life [4-11]. 

The Social Security Amendments of 1965 established the Medicaid program, which was signed into law by President Lyndon B. Johnson to expand healthcare access and reduce insurance inequities across socioeconomic, racial, and ethnic groups [12]. Medicaid has since become the largest health insurance program by enrollment in the United States, providing coverage for low-income individuals across diverse populations of children, adults, pregnant individuals, older adults, people with disabilities, and racial and ethnic minorities [13]. In 2022, Medicaid covered an average of 80.6 million individuals (24.2% of the population) each month and accounted for an estimated $671.2 billion in health expenditures in 2020 (16.3% of national healthcare spending) [13]. Given Medicaid’s pivotal role in expanding access to vision care for underserved populations, provider participation among both ophthalmologists and optometrists is essential to ensure equitable and timely care for enrollees.

The scope of practice for optometrists is determined by each state’s legislature and varies considerably among states [14-19]. Over the past two decades, states such as Oklahoma, Kentucky, and Louisiana have passed legislation that expands optometrists’ scopes of practice, and current legislative efforts in several additional states seek surgical privileges [17-21]. Some legislators argue that eye care access remains inadequate in certain regions and that expanding optometrists’ scope of practice may alleviate perceived gaps in service delivery [19].

For example, during the 2017 legislative hearings for Florida House Bill 1037, optometrists attempted to justify scope expansion by arguing that only 700 ophthalmologists in the state accepted Medicaid. Subsequent analyses by the Florida Society of Ophthalmology (FSO) and the American Academy of Ophthalmology (AAO), however, identified over 1,400 ophthalmologists enrolled in Medicaid and reported that ophthalmologists participated in the Medicaid program at higher rates than optometrists. Because of these conflicting claims, the present study was performed to better understand access to Medicaid-covered eye care in Florida by comparing ophthalmologists and optometrists with respect to program participation, willingness to accept new Medicaid patients, and appointment availability.

## Materials and methods

A cross-sectional survey study was conducted to assess Medicaid access to ophthalmologists and optometrists practicing in the state of Florida. This study adhered to the tenets of the Declaration of Helsinki and complied with regulations of the Health Insurance Portability and Accountability Act. All survey response data was deidentified. Institutional review board review approval was waived since no patient data was included in this study. 

A complete list of ophthalmologists and optometrists registered with Florida Medicaid, along with their demographic data (sex, age, race, and ethnicity), was compiled. All data were extracted from the Florida Agency for Health Care Administration database [22,23]. A geographically uniform sample of 22 ophthalmologists and 80 optometrists from this list was randomly selected and contacted via telephone between February 2018 and March 2018. Zip codes that were used to determine the county of each provider’s primary office were then matched with their corresponding United States Department of Agriculture’s Urban Influence Code (UIC) [24]. The UIC scores range from 1 to 12, with codes 1 to 3 categorized as 'urban' for the purpose of this study.

**Figure 1:**
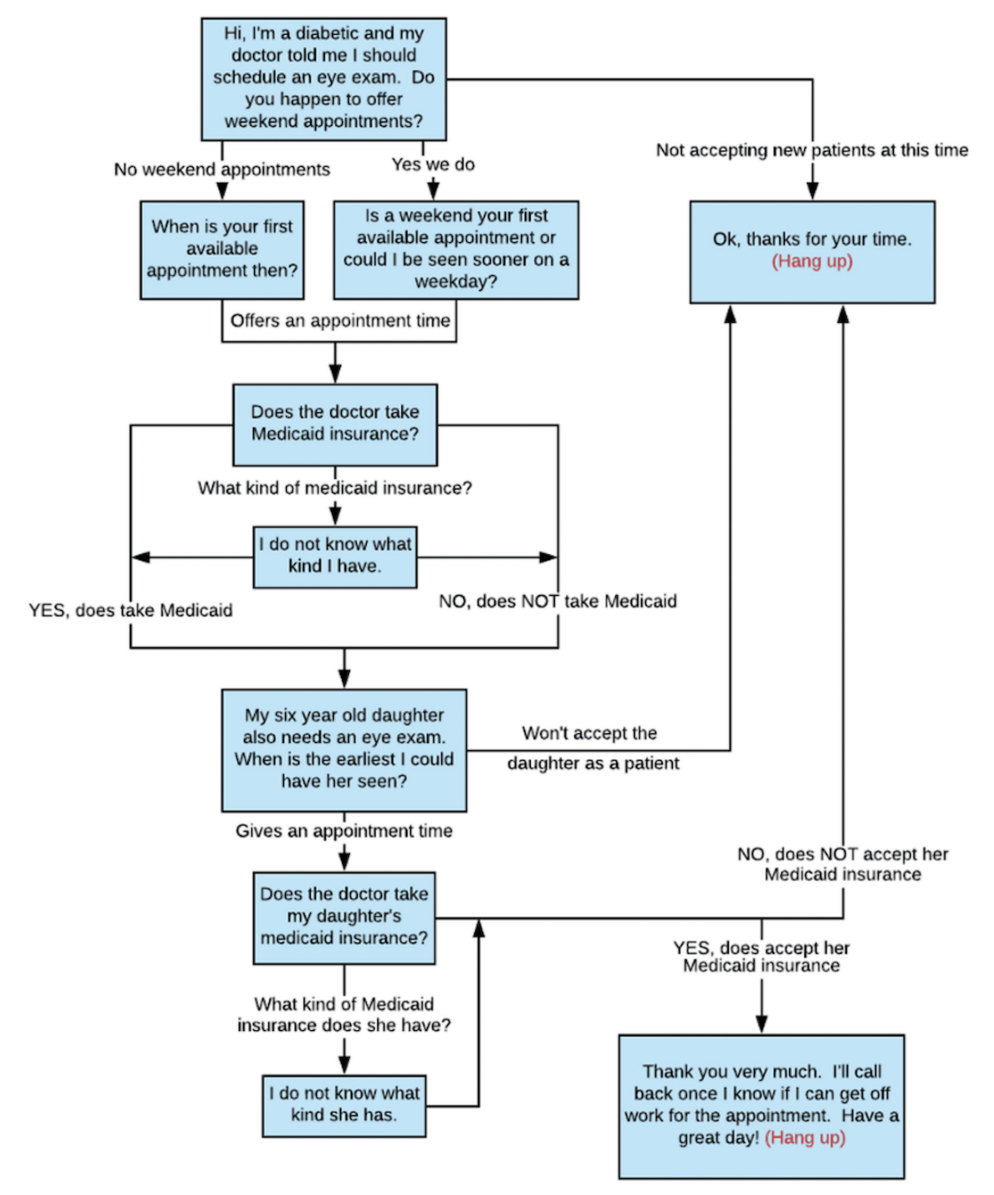
Caller script for each provider Image credit: Authors Miller and Lee

Trained telephone interviewers used a standardized script with the same hypothetical patient scenario to ensure consistency in survey delivery and data acquisition. The anonymously conducted survey took the form of an inquiry from a potential new patient seeking care. During each telephone call, provider offices were asked a series of questions to determine if they accept new Medicaid patients, if they accept new adult and/or pediatric Medicaid patients, and their next available appointment date from the time of the call. The script can be found in Figure 1. All calls were made on weekdays between 9:00 a.m. and 5:00 p.m. local time, and if an office could not be reached after three contact attempts, no further efforts were made, and a substitute provider was randomly selected.

Statistical analyses were performed by the Department of Quantitative Health Sciences at Mayo Clinic Jacksonville (FL, USA). Categorical survey answers were described as frequencies (percentages), and continuous survey answers were presented as descriptive statistics (median and range). Categorical responses were compared between ophthalmologists and optometrists using Fisher’s exact test, and the number of days to the next available appointment was compared using the Wilcoxon rank-sum test. All statistical tests were two-sided, with a significance level set at α=0.05.

## Results

A comparison of survey responses between ophthalmologists and optometrists is presented in Table 1. Across all age groups, a greater proportion of ophthalmologists than optometrists reported accepting some form of Medicaid (95.4% vs. 83.8%; p=0.29). Similarly, higher percentages of ophthalmologists indicated they accepted new Medicaid patients (86.4% vs. 76.3%; p=0.39) and adult patients with Medicaid coverage (90.9% vs. 85.0%; p=0.73). Acceptance rates for new pediatric Medicaid patients were similar between ophthalmologists and optometrists (46.3% vs. 45.5%; p=1.00). Weekend availability was also comparable, with 63.6% of ophthalmologists and 56.3% of optometrists offering appointments (p=0.63), and the median time to the next available appointment was nine days for ophthalmologists and eight days for optometrists (p=0.74). No statistically significant differences between ophthalmologists and optometrists were identified in any survey response. 

**Table 1:** Survey answers of ophthalmologists and optometrists OD: Optometrist; MD: Ophthalmologist; Q: Quartile

Category	OD (n=80)	MD (n=22)	Total (n=102)	p-value
Urban location				0.1480
No	41 (51.3%)	7 (31.8%)	48 (47.1%)	
Yes	39 (48.8%)	15 (68.2%)	54 (52.9%)	
Accepts new adults				0.8021
No	12 (15.0%)	2 (9.1%)	14 (13.7%)	
Yes	34 (42.5%)	11 (50.0%)	45 (44.1%)	
Accepts some forms of Medicaid but not all	34 (42.5%)	9 (40.9%)	43 (42.2%)	
Accepts new adults				0.7286
No	12 (15.0%)	2 (9.1%)	14 (13.7%)	
Yes, or some forms of Medicaid, but not all	68 (85.0%)	20 (90.9%)	88 (86.3%)	
Accepts new children				1.0000
The doctor only sees adults	11 (13.8%)	3 (13.6%)	14 (13.7%)	
No	32 (40.0%)	9 (40.9%)	41 (40.2%)	
Yes	37 (46.3%)	10 (45.5%)	47 (46.1%)	
Accepts new patients				0.3907
No	19 (23.8%)	3 (13.6%)	22 (21.6%)	
Yes	61 (76.3%)	19 (86.4%)	80 (78.4%)	
Weekend availability				0.6294
No	35 (43.8%)	8 (36.4%)	43 (42.2%)	
Yes	45 (56.3%)	14 (63.6%)	59 (57.8%)	
Days to appointment				0.7431
N	54	19	73	
Median	8.0	9.0	9.0	
Q1, Q3	4.0, 14.0	3.0, 16.0	3.0, 16.0	
Range	(1.0-275.0)	(1.0-305.0)	(1.0-305.0)	

## Discussion

In this cross-sectional survey study, a greater percentage of ophthalmologists accepted some form of Medicaid, provided care to new adult Medicaid patients, and offered weekend appointment availability compared to optometrists, with comparable wait times for the next available appointment across both provider types. Although these differences did not reach statistical significance, these observed trends challenge the assertion made by proponents of optometric scope expansion that ophthalmologists in Florida offer limited accessibility for Medicaid patients. As policymakers evaluate proposals to expand the optometric scope of practice to include surgical procedures, they should consider the multifaceted nature of access to care that includes not only geographic proximity of the providers but also their insurance participation and appointment availability. 

Evidence comparing Medicaid participation between ophthalmologists and optometrists is limited and inconsistent, with no comprehensive studies conducted within the past two decades. A 1989 national survey of 1,003 ophthalmologists and 1,000 optometrists across the United States private practices reported comparable rates of Medicaid acceptance (51% vs. 55%, respectively) [25]. A 1990 survey of 200 ophthalmologists and 334 optometrists in Oregon found that only 11% of ophthalmologists accepted Medicaid (compared to 70% of optometrists) [26]. Whereas a 2004 survey of 364 pediatric-based eye care practices in Michigan found a higher Medicaid acceptance rate among ophthalmology-listed practices (74% vs. 59%, p<0.01) [27]. In our Florida-based study, Medicaid acceptance was markedly higher across both provider groups compared to prior reports (ophthalmologists: 95.4%; optometrists: 83.8%), though these results may reflect regional variability and the study’s limited sample size. 

The availability of appointments with each type of provider further complicates this discussion. A 2018 study of 303 ophthalmologists and 300 optometrists in Maryland and Michigan found that Medicaid-enrolled pediatric and adult patients were significantly more likely to obtain an appointment with an optometrist (pediatric: OR, 8.00; 95% CI, 5.37-11.90; p<0.001; adult: OR, 1.91; 95% CI, 1.31-2.79; p<0.001) though Medicaid participation rates by provider types were not reported [28]. In contrast, we found minimal differences in access metrics, including weekend availability (ophthalmologists vs. optometrists, 63.6% vs. 56.3%) and median wait time to next available appointment (nine vs. eight days, respectively), suggesting that the perceived ease of access to optometrists may be overstated in some settings.

Provider proximity remains another important factor in assessing the impact of scope-of-practice expansion on access to care, with previous efforts having relied on Medicare claims data [17,29-31]. The largest analysis to date evaluated 1.56 million claims from 2016 to 2020 in four states that permit optometric laser surgery privileges (Arkansas, Kentucky, Louisiana, and Missouri) [19]. Expanded optometric privileges did not lead to reduced travel times or increased population-level access to eye care professionals, raising questions about the effectiveness of scope expansion as a solution to geographic disparities in care [19]. 

The above discussion is centered on insurance coverage and clinic availability, but the quality of care, too, remains an equally important consideration. Ophthalmologists undergo substantially more specialized training in performing intraocular injections, eyelid surgeries, and laser procedures, and this may result in better procedural outcomes. An analysis of Medicare claims in Oklahoma found that patients with glaucoma who were treated by optometrists had a 189% higher likelihood of requiring repeat laser trabeculoplasty in the same eye compared to those treated by ophthalmologists (35.9% vs. 15.1%; hazard ratio (HR), 2.89; 95% CI, 2.00-4.17; p<0.001), indicating differences in provider expertise and efficacy of the procedures [32]. Our study was not designed to directly evaluate provider proximity or quality measurements, but these metrics should be considered during policy discussions over optometric scope expansion.

This study has several strengths and weaknesses. The small sample size in this pilot study limits the generalizability of these findings, but a larger, more comprehensive study is planned to address this. State-associated variability in Medicaid insurance coverage further limits the applicability of these results to other regions within the United States [33]. These data indicate a trend toward higher acceptance of Medicaid and adult patients by ophthalmologists in Florida, but these findings should be interpreted with caution since differences in survey responses failed to reach statistical significance. This analysis did not account for structural and social determinants of healthcare access, such as language barriers, transportation challenges, and inflexible work schedules, all of which limit the broader implications of these findings. 

## Conclusions

To our knowledge, this is the first study to evaluate differences in Medicaid accessibility between ophthalmologists and optometrists exclusively within the state of Florida. Furthermore, it is the only such analysis conducted in this region in the past two decades. These findings add important context to ongoing national discussions regarding optometric scope-of-practice expansion and challenge the narrative that ophthalmologists offer limited access to Medicaid patients.
